# Agent-Based Model Forecasts Aging of the Population of People Who Inject Drugs in Metropolitan Chicago and Changing Prevalence of Hepatitis C Infections

**DOI:** 10.1371/journal.pone.0137993

**Published:** 2015-09-30

**Authors:** Alexander Gutfraind, Basmattee Boodram, Nikhil Prachand, Atesmachew Hailegiorgis, Harel Dahari, Marian E. Major

**Affiliations:** 1 Division of Epidemiology and Biostatistics, School of Public Health, University of Illinois at Chicago, Chicago, Illinois, United States of America; 2 The Program for Experimental & Theoretical Modeling, Department of Medicine, Loyola University Medical Center, Maywood, Illinois, United States of America; 3 Center for Biologics Evaluation and Research, Food and Drug Administration, Silver Spring, Maryland, United States of America; 4 STI/HIV Surveillance, Chicago Department of Public Health, Chicago, Illinois, United States of America; 5 Department of Computational Social Science, George Mason University, Fairfax, Virginia, United States of America; 6 Theoretical Division, Los Alamos National Laboratory, Los Alamos, New Mexico, United States of America; University Medicine Greifswald, GERMANY

## Abstract

People who inject drugs (PWID) are at high risk for blood-borne pathogens transmitted during the sharing of contaminated injection equipment, particularly hepatitis C virus (HCV). HCV prevalence is influenced by a complex interplay of drug-use behaviors, social networks, and geography, as well as the availability of interventions, such as needle exchange programs. To adequately address this complexity in HCV epidemic forecasting, we have developed a computational model, the Agent-based Pathogen Kinetics model (APK). APK simulates the PWID population in metropolitan Chicago, including the social interactions that result in HCV infection. We used multiple empirical data sources on Chicago PWID to build a spatial distribution of an *in silico* PWID population and modeled networks among the PWID by considering the geography of the city and its suburbs. APK was validated against 2012 empirical data (the latest available) and shown to agree with network and epidemiological surveys to within 1%. For the period 2010–2020, APK forecasts a decline in HCV prevalence of 0.8% per year from 44(±2)% to 36(±5)%, although some sub-populations would continue to have relatively high prevalence, including Non-Hispanic Blacks, 48(±5)%. The rate of decline will be lowest in Non-Hispanic Whites and we find, in a reversal of historical trends, that incidence among non-Hispanic Whites would exceed incidence among Non-Hispanic Blacks (0.66 per 100 per years vs 0.17 per 100 person years). APK also forecasts an increase in PWID mean age from 35(±1) to 40(±2) with a corresponding increase from 59(±2)% to 80(±6)% in the proportion of the population >30 years old. Our studies highlight the importance of analyzing subpopulations in disease predictions, the utility of computer simulation for analyzing demographic and health trends among PWID and serve as a tool for guiding intervention and prevention strategies in Chicago, and other major cities.

## Introduction

HCV is a major public health threat with 130–150 million chronic cases worldwide [1], including 2.7–3.9 million in the U.S. [2], and is the leading cause of cirrhosis, liver cancer, liver failure, and liver transplantation [3]. In the U.S., mortality related to viral hepatitis exceeded that for human immunodeficiency virus (HIV) between 1999 and 2007 [4]. Every year, 7,000–43,000 new cases of HCV infection are estimated to occur in the U.S. [2] and 2–4 million new cases worldwide [5]. In developed countries, the primary mode of HCV transmission is illicit drug use [6], with an estimated 60% of all HCV infections in the U.S. attributable to drug injection [7].

The injecting drug population in the U.S. has been experiencing a long-term demographic shift that impacts HCV transmission and prevalence in complex ways. For at least a decade, multiple studies in Chicago, Illinois and other areas have documented a shift in the racial/ethnic composition of the U.S. drug-user population [8–10]. These data consistently show that young persons initiating into injection drug use are increasingly likely to be non-Hispanic Whites from suburban communities rather than the urban, low-income minority populations that have typified people who inject drugs (PWID) since at least 1950 [9]. These studies are further supported by high incidence of HCV infection [11] and outbreaks among younger PWID [12–14] while HIV [15] and HCV [16] prevalence have steadily declined in older populations. Factors contributing to HCV infection acquisition and successful intervention among PWID are complex and occur at the individual, social and geographic levels (e.g., risk behaviors, injection networks and interaction locations, respectively). The impact of these trends on HCV epidemics over time has not been adequately considered in forecasting and planning.

Dynamic modeling is a useful method for simultaneously accounting for the aforementioned complexities inherent in forecasting HCV transmission and prevalence among PWID and can be divided into two main categories—Compartmental Models (CM) and Agent-Based or Individual-Based Models (ABMs/IBMs) [17]. Compartmental models are the most commonly used class for modeling HCV epidemics among PWID [17]. The CM approach is usually a set of differential equations that assign the PWID population into compartments based on their HCV infection state (e.g. susceptible, infected, recovered), along with other attributes such as enrollment in antiviral/vaccine treatment, harm reduction programs, or imprisonment [18,19]. For simplification, these models consider homogenous populations of PWID within a given compartment although it is known that PWID populations are highly heterogeneous. The ABMs/IBMs approach addresses this limitation and offers a more realistic modeling strategy that accounts for the variability of individual characteristics and behaviors [20]. These models may include factors anticipated to vary between individuals based on empirical studies, including injection network [21–23], age, length of time since initiating injection drug use, injection behaviors (e.g. sharing of injection equipment) and geographic locations [24–27]. ABMs have been previously applied to study HCV infections among PWID [28–31] and other infections such as influenza [32–34].

In the current study, we applied the ABM methodology [20] to study the approximately 32,000 PWID who reside in metropolitan Chicago, IL, U.S. [35] and undertook to forecast HCV prevalence among this group. Our software is termed the Agent-based Pathogen Kinetics model (APK) and it represents a novel computational modeling platform. In APK, each PWID performs drug-related daily activities, has a state of HCV infection (if infected), location of residency and could maintain a drug sharing network with other PWID. APK was designed using highly-detailed empirical networks and geospatial Chicago-based data from large epidemiological empirical datasets to model the PWID population in each neighborhood; capturing associations between demographic characteristics (e.g. age, race/ethnicity) and drug use risk behaviors.

The main novelty of APK is the detailed simulation of four aspects of the drug lifestyle: demographic, behavioral, social and geospatial. Whereas previous studies introduced social networks and behavioral simulations, few have explored how they are influenced by geographic and demographic processes [17]. In addition, APK is based on unique multi-annual surveys of the Chicago drug epidemic, which have not been previously studied using agent-based models [17].

We used APK to forecast long-term trends in HCV incidence and prevalence among PWID in metropolitan Chicago. We validated the forecast with empirical data and predict a number of trends over the next decade, including aging of the PWID population and the persistence of high HCV prevalence among certain sub-groups of PWID.

## Materials and Methods

### Epidemiological and Network Datasets

Four datasets were used for these studies that contained information from surveys performed with people who inject drugs. All data were analyzed anonymously. Three of these studies have been published previously. All study procedures for the Needle Exchange Program and Young Social Networks studies were approved by the Institutional Review Board of the University of Illinois at Chicago and the use of the NHBS data was approved through a data agreement with the Chicago Department of Public Health.

**Fig 1:**
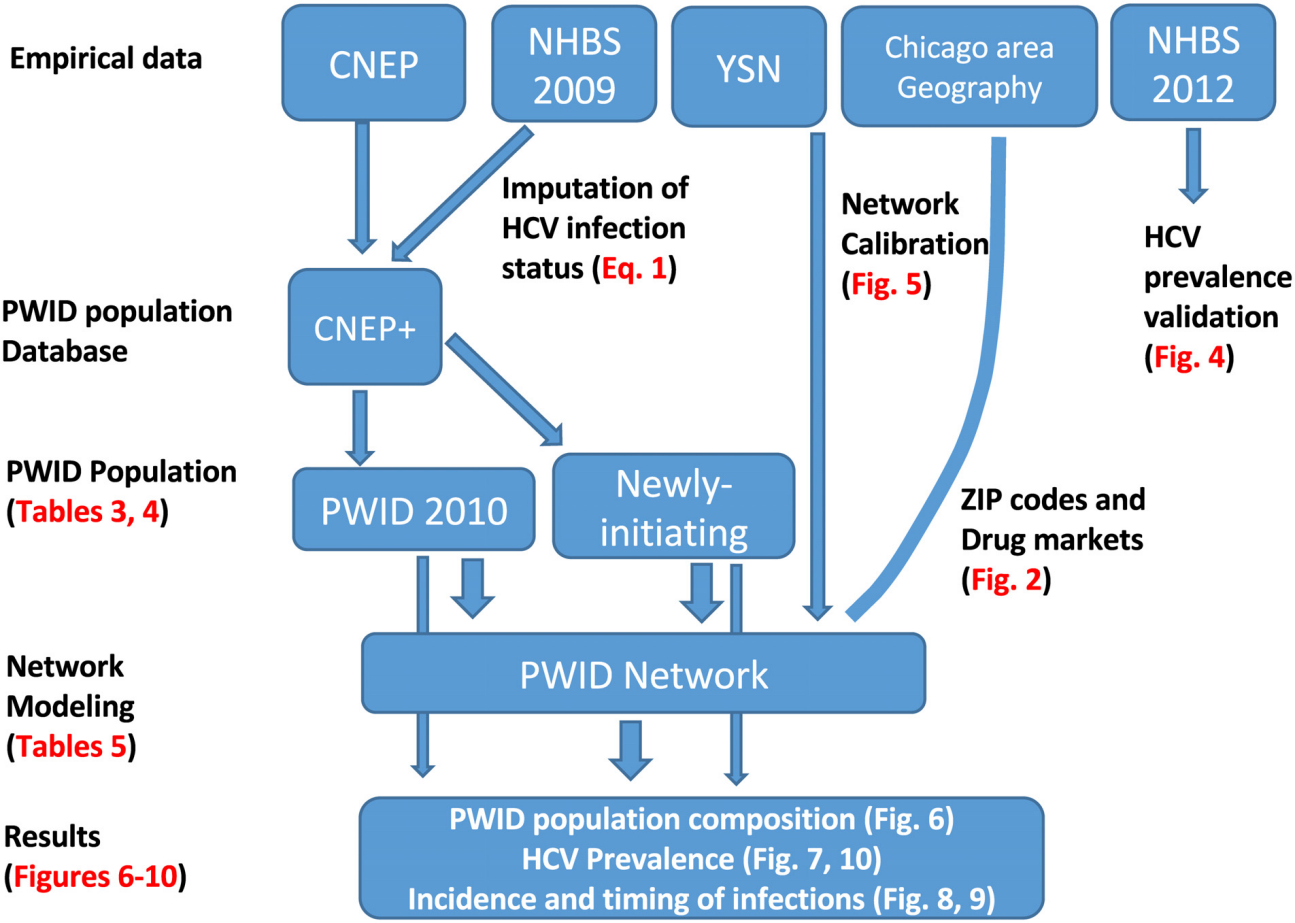
Schematic representation of APK design. The empirical data is represented by five main datasets. The incorporation of each dataset in the development of APK is shown by arrows. The locations of specific data outputs in the form of Tables and Figures are shown in Red type. CNEP: Community Outreach Intervention Projects (COIP) Needle Exchange Program (NEP); NHBS: National HIV Behavioral Surveillance; YSN: Young Social Networks; CNEP+: Enhanced CNEP population generated for APK.

The five main datasets used in developing APK are described below; the use of each dataset in the development is shown in Fig 1.

#### The COIP Needle Exchange Program (CNEP) Survey

The base dataset for APK came from the ongoing Community Outreach Intervention Projects (COIP) Needle Exchange Program (NEP) that operates at four Chicago storefront locations and at multiple urban locations through a mobile outreach unit. COIP has provided harm reduction supplies such as sterile syringes/needles and counseling, as well as collecting extensive research data on metropolitan Chicago PWID since 1996. About 1,500 registered PWID regularly visit one of COIP’s storefront locations or mobile units each year. The ongoing COIP Needle Exchange Program (CNEP) survey [36] collects data at the enrollment visit of each participant, including demographic characteristics, place of residence, injection drug use practices and behaviors, and health status. We accessed more than 6,000 CNEP questionnaires collected from 2006–2013 from clients residing throughout the Chicago metropolitan area. The large size and wide geographic coverage of the NEP provides the most comprehensive data on the metropolitan Chicago PWID population and was used to map the distribution of PWID.

#### The CDC-sponsored National HIV Behavioral Surveillance (NHBS) survey for Chicago (years 2009 and 2012)

Through a data agreement with the Chicago Department of Public Health, we obtained the NHBS surveys for 2009 and 2012 specific to metropolitan Chicago PWID. In the 2009 survey [37], 545 eligible PWID were enrolled using respondent-driven sampling, a type of chain referral sampling that has been shown to be effective in finding hidden populations such as PWID who may live across a wide geographic area [38]. The interviews were conducted between August 12, 2009 and November 24, 2009. NHBS includes data on HCV risk and HCV antibody prevalence. The NHBS 2012 (n = 209) applied the same methodology as the NHBS 2009 between October 18, 2012 and December 21, 2012. We used the 2009 HCV antibody prevalence data as an estimate of the HCV prevalence at the start of our simulation (January 1, 2010) and the 2012 NHBS HCV antibody prevalence to validate the simulation.

#### The Young PWID Social Networks Study

The Young Social Networks survey (2013) is an unpublished study led by the Division of Epidemiology and Biostatistics, School of Public Health, UIC, Chicago, IL (Boodram, personal communication) which collected information regarding demographic characteristics (e.g. age, race/ethnicity), place of residence(s), injection related risk practices (e.g. sharing needles), and self-reported HIV and HCV status data on 164 young (ages 18–30 years) PWID and the people they injected drugs with most frequently (core network) in the prior 6 months. We used these data to calibrate and validate the construction of APK’s social risk network.

#### Geography data

**Fig 2:**
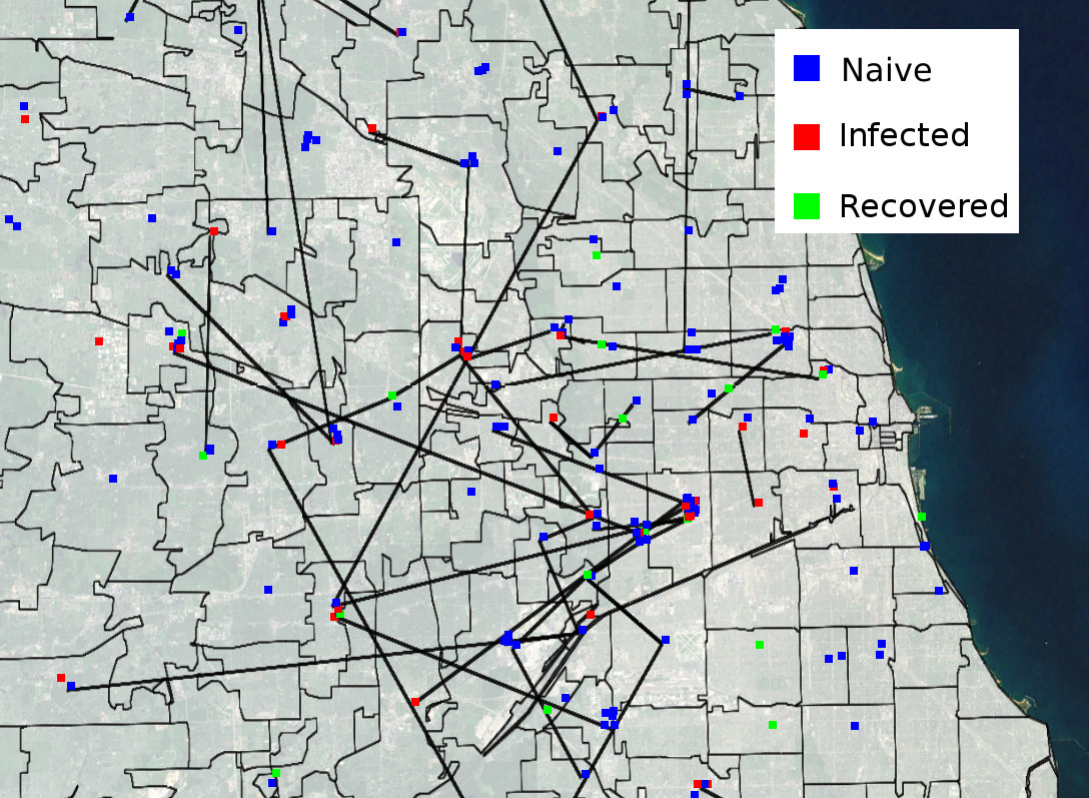
APK screen showing the Chicago metropolitan area. The screen shows people who inject drugs (PWID, small squares), geographic zones (gray regions) and relationships (straight black lines). PWID are colored based on their hepatitis C status: Red—HCV- Infected, Blue—naïve, and Green—HCV-recovered. For clarity, this simulation displays just 320 agents, 1% of the entire APK PWID population. Orange circles—major drug markets.

The Chicago metropolitan area has an area of 28,120 km^2^ and had a population of 9.7 million in 2010. The City of Chicago had a 2010 population of 2.7 million and can be divided into three large areas with distinct demographics—the North, West and South sides. The metropolitan Chicago area is represented in APK with over a hundred geographic zones using postal (ZIP) codes from the 2010 U.S. Census. The location of drug markets was based on multiple data sources from COIP researchers [24,39–41]. Fig 2 shows the geographic layout of a subset (1%) of these data as a screen shot of the APK PWID in the city and connections between them based on locations.

### HCV Infection Stages

**Fig 3:**
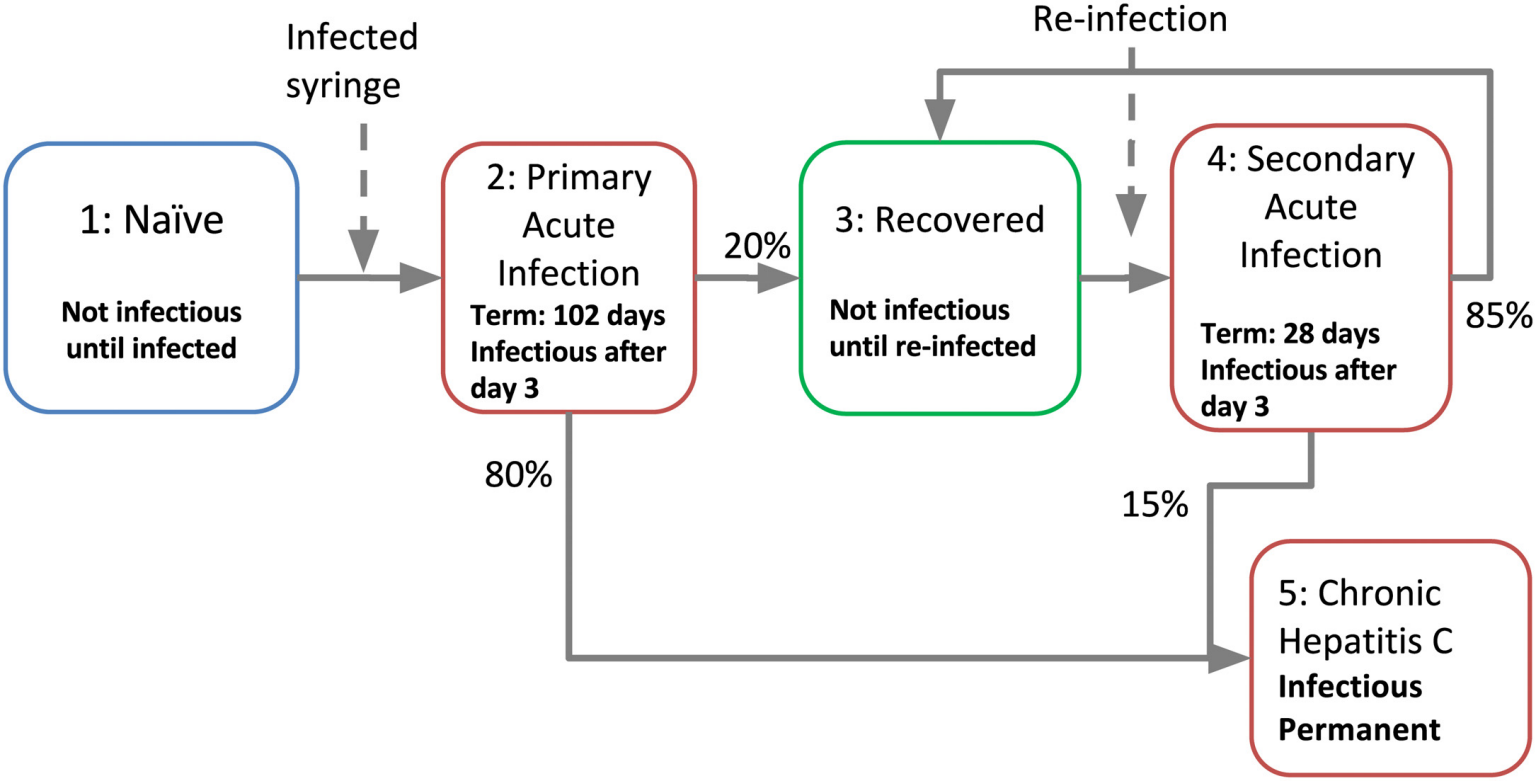
Stages in the progression of infection with HCV. The duration of each stage is indicated (if temporary) together with the probability of transition to another state of HCV infection. Probabilities are shown as representative values (details in Table 1).

Each *in silico* individual may be at any given time in one of the following HCV infection stages (Fig 3): (1) naïve (or susceptible), (2) primary acute, (3) recovered phase (spontaneously cleared the virus until re-infected), (4) non-primary acute phase (i.e., re-infected), and (5) chronic viremic phase (life-long). Primary acute infection (Stage 2) usually results in chronic life-long infection (Stage 5), but about 20% of cases experience spontaneous recovery (Stage 3). If an individual spontaneously clears HCV during the acute stage (recovers) then some natural immunity is attained, which leads to a modified infection upon re-exposure [42,43]. Recovered individuals have improved outcomes if they acquire HCV again, including shorter duration of acute infection (Stage 4) and a lower risk of developing chronic HCV infection. HCV infection parameters, values and sources [30, 42–46] for these values are shown in Table 1. We based the values for duration of HCV infection (primary and secondary infections) and clearance rates on empirical data obtained from our meta-analysis of 99 chimpanzees [42], which have been shown to be comparable to data from human studies [43].

**Table 1:** HCV infection parameters.

Parameter	Description	Value	Range	Refs
mean_days_acute_naive	Duration of acute infection in a naïve individual	102	77–127	[42,44]
mean_days_acute_rechallenged	Duration of acute infection in a recovered individual	28	8–48	[42]
mean_days_naive_to_infectious	Time taken for an infected individual to become infectious	3	2–4	[42]
prob_clearing	Probability of a recovered individual clearing virus upon re-exposure^a^	0.85	0.75–0.95	[42,43]
prob_self_limiting_female	Probability of spontaneous viral clearance upon first exposure- females	0.346	0.30–0.40	[45]
prob_self_limiting_male	Probability of spontaneous viral clearance upon first exposure—males	0.121	0.10–0.14	[45]
transmissibility	Probability that a naive PWID will be infected in a receptive sharing event with an infected PWID	0.01	0.0005–0.05	[30,46]

^a^, regardless of gender

### Generation of the Synthetic Population

We calculated that approximately half of the estimated 32,000 PWID [35] in metropolitan Chicago are similar to those enrolled in harm reduction programs. We used the large CNEP dataset (n~6,000) to construct the harm reduction-based population and developed an algorithm (described in S1 Text) to construct the non-harm reduction-based population. Briefly, since we expect harm reduction and non-harm reduction to be demographically similar [36], we constructed the non-harm reduction profiles based on CNEP, but adjusted for reported differences [36] in risky behaviors and health status. The resulting synthetic PWID population (termed) CNEP+ was used to generate the *in silico* initial population, i.e. the population on January 1, 2010. We expect that the non-parametric process (i.e. population modeling process that draws from a large database) represents the spatially complex and internally correlated structure of the PWID population, including e.g. correlations of age and race, with greater accuracy than alternatives based on parametric models (e.g. [28]). The parameters used in this process are shown in Table 2 based on [42, 47–50]. The values of the parameters and the range of values were rigorously reviewed by the team of co-authors that include an epidemiologist, virologist, and hepatologist against additional empirical studies in their respective areas of expertise to align with literature beyond the scope and breadth of the datasets.

**Table 2:** Parameters for the generation of the synthetic population.

Parameter	Value	Range	Notes
ab_prob_acute^a^	0.02	0.01–0.03	set to 2% based on relative durations of acute phase and injection career
ab_prob_chronic^a^	0.67	0.49–0.74	[42,47]
attrition_rate	0.024 per year	0.01–0.08	[48]
burn_in_days	365	-	Calibrated by observing the time necessary for the HCV incidence to stabilize.
initial_PWID_count	32,000	30,000–34,000	[49]
mean_career_duration (years)	30.3	10–35	[48]
prob_cessation	0.232	0.13–0.33	[48]
prob_infected_when_arrive (acute HCV infection)	0.05	0.01–0.09	0.9% prevalence among newer PWID, and 9% among other* PWID [50]
PWID_maturity_threshold (years)	5	3–7	Set by trading off sample size (larger value) and accuracy (smaller value).

^a^, 1-(ab_prob_acute+ab_prob_chronic) represents the probability of HCV antibody positive PWID who is not viremic (i.e., recovered).

*, PWID who acquired HCV prior to initiating into injection drug use through other modes (e.g. sharing non-injection drug paraphernalia such as snorting straws.

At the start of the simulation, the population consists of the approximately 32,000 PWID (parameter **initial_PWID_count**), which are drawn from the CNEP+ data. New PWID (i.e. newly-initiating) enter the population over 2010–2020 and they represent those who are beginning injection drug careers. We expect that these individuals would be subtly different from the CNEP+ population since the existing population represents a wider range of injection career histories. To account for these differences, we modeled the newly-initiating PWID using a non-parameteric data-driven approach, i.e. newly-initiating PWID are patterned after PWID in CNEP+ who have been engaging in injection drug use for a relatively short time (e.g. < 5 years) (parameter **PWID_maturity_threshold**). We assume that the majority of the newly-initiating PWID are HCV-uninfected since they enter the simulation when they initiate into injection drug use. However, we assigned 5% of the newly-initiated PWID as acutely infected (parameter **prob_infected_when_arrive**) to account for other modes of HCV acquisition, which may include sharing non-injection equipment with others prior to initiating into injection drug use [51].

In APK, PWID can leave the simulation due to either cessation of injection drug use (parameter **prob_cessation**), or continue injection drug use until death or incarceration. Those who achieve cessation are assigned a career duration from a normal distribution (parameter **mean_career_duration)** of approximately 30 years based on published empirical studies and a standard deviation of 12 years [48].

The annual probability of death increases with age while incarceration decreases with age [48]. Because the effects compensate each other, we characterized the combined attrition due to death and incarceration using an exponential attrition rate (parameter **attrition_rate)**.

When the simulation is initiated, there is a tendency for the prevalence of HCV to fluctuate due to the formation of many discordant relationships (not shown). To prevent this from biasing the results, we used a burn-in procedure that continues for 365 simulated days (parameter **burn_in_days)**. During the burn-in PWID neither enter nor leave the simulation, nor do they grow older, but have ongoing HCV transmission and network dynamics. We initiate data collection after the burn-in period and denoted this time point as January 1^st^, 2010.

### Initial HCV infection state

We applied a logistic regression classification approach (Eq 1) to assign HCV status in each individual in CNEP+.

$$L\left(X\right)=\frac{\text{exp}\left(-\sum_{i}\beta_{i}X_{i}\right)}{1+\text{exp}\left(-\sum_{i}\beta_{i}X_{i}\right)}$$ (1)

Where X is demographic information about the PWID in CNEP+ and L is the logistic link function. To train the classifier, the following information is read from NHBS 2009: age, age of first injecting drug use, size of drug in-network, gender, race, ZIP code of residence, frequency of injections per day, probability of receptive sharing, and the HCV antibody status. In 10-fold cross validation (i.e., when 1/10 of the data is left out of the calibration and instead used for testing), the classifier achieved an average receiver operating characteristic (ROC) score of 0.68 (maximum is 1.0). All imputations were performed using the Weka machine learning toolkit version 3.6.11 [52].

When presented with a profile from CNEP+, the classifier produces a probability that the person is in a particular HCV AB status (AB+ or AB-). If an individual is AB+ three HCV infection states are possible: acute, chronic (i.e., viremic), or recovered (no virus). These infection states are assigned based on the probabilities in Table 2. We calculated that approximately 2% of the CNEP+ population are acutely-infected (parameter **ab_prob_acute**), 67% are chronically infected (parameter **ab_prob_chronic)** and the remainder are recovered (31%). The parameter **ab_prob_acute** is calculated from the observation that the duration of the acute phase is approximately 100 days and represents less than 2% of the duration of a 10-year PWID career and less than 1% of a 20-year PWID career.

### Attributes

Each PWID in the simulation has attributes, i.e. quantities that vary from individual to individual such as location of residence, race/ethnicity, gender, frequency of drug injection, and HCV infection status. These attributes are shown in Table 3 with representative statistics for the start of the simulation in 2010. For PWID in 2010 (the initial population), the elapsed years of injection drug use represents the amount of time a subject has been injecting drugs at this time point in the simulation. Thus, the average elapsed time for injecting drug use for PWID within APK is 11.4 years in 2010. This is distinct from the mean career duration of 30 years used to generate the synthetic population (Table 2), which represents the total length of time for an individual to reach cessation of drug use, i.e. the total length of an individual’s injecting career. The simulation also maintains information about the network of relationships among PWID, which we refer to as the social drug risk network. The network is directed in that each relationship has the attributes from-PWID and to-PWID. The from-PWID (or giver) shares drugs, injection equipment with the to-PWID (the recipient). The PWID’s in-degree and out-degree attributes (Table 3) specify the number of receiving and giving relationships, respectively. New infections with HCV may occur among PWID with in-degree ≥1 who are exposed to contaminated equipment (i.e., blood transmission from viremic PWID).

**Table 3:** Attributes of PWID and representative data.

Attribute	Statistics for 2010
*Demographic attributes*	
Location (by ZIP code)	City: 46%; Suburbs: 54%
Race/ethnicity	Hispanic: 18% NH Black: 21% NH White: 58% NH Other: 3%
Gender	Female: 30%; Male: 70%
Age	Mean: 35.3 years. IQR: 26.1–43.0 Over 30: 59% Under 30: 41%
Elapsed years of injection drug use	Mean: 11.4 years. IQR: 3.3–16.0
Enrollment in any HR program	HR: 48%; non-HR: 52%.
HCV infection state	Infected (acute or chronic): 30% Recovered (antibody +): 13%
*Behavioral attributes*	
Daily drug injections	Mean: 2.5; IQR: 0.89–3.26
Probability of receptive sharing Ranges from 0 (never) to 1 (every injection)	Mean: 19%*. IQR: 0.0%-37%
*Network attributes*	
In Degree (receptive network size)	56%- 0 (no network), 32%- 1, 12%- ≥2
Out Degree (giving network size)	65%- 0 (no network), 25%- 1, 10%- ≥2

NH = Non-Hispanic. IQR = interquartile range. HR = harm reduction program.

*i.e. sharing or equipment reuse in 19% of injections while 81% of injections involve sterile equipment.

Each *in silico* PWID performs a finite number of drug injections every day, which is represented by a Poisson distribution around an individual mean value (Table 3). The probability of receptive needle sharing is an attribute of the individual, and varies across the population (Table 3). To represent these sharing events, the simulation selects at random a person from the PWID’s in-network partners for sharing. The probability that a naive PWID would be infected is represented by the parameter **transmissibility** (Table 1).

The CNEP dataset indicates that drug behavior remains stable over the PWID injection career (e.g.<1% relative change per year of career in number of partners, number of daily injections or probability of sharing). As such, we assume in APK that once an *in silico* individual is assigned a drug behavior it remains as is during her/his injecting career.

### Network Formation

A central calculation for network formation is to determine the probability that two persons are likely to encounter each other and form a relationship that promotes HCV transmission. APK allows for three kinds of encounters (1) in the neighborhood close to the place of residence; (2) at a drug purchase market, and (3) other encounters, which includes co-workers or any stranger in the city. The methods used to calculate network encounter rates, establishment processes, and removal of networks are detailed in S1 Text (Supplemental Methods).

The network in the simulation is the sole route of transmission for HCV. Some individuals who enter the simulation and who are assigned a network rapidly create a number of injecting-related contacts (or “ties”). In these subjects, the network is dynamic, and during the course of their career, some ties may be lost, while new ones form but result in an approximately constant network size. We used the CNEP+ and NHBS 2009 data to determine the initial size of injection network for each PWID in the synthetic population based on the premise that sharing of drugs, needles or paraphernalia transmits HCV [26,30]. The CNEP data also suggests that PWID may have two distinct groups of partners, and the sizes of the two groups may be very different [36]: the in-network (termed in-degree, Table 3) partners are people giving drugs or equipment to the PWID, while the out-network (termed out-degree, Table 3) are those receiving drugs or equipment from the PWID. For this reason, each PWID in APK maintains both kinds of partnerships, with some overlap.

The network construction is informed by the geography (based on ZIP codes) and the homophily for the person’s race/ethnicity and age (Table 4). The network changes over the course of the simulation to represent the turnover in the PWID population, that is, the creation and dissolution of social relationships. We assigned each PWID to a region defined by a single postal ZIP code, which represents the PWID’s place of residence or the place of sojourn for homeless individuals. We used each PWID’s ZIP code to determine his or her probability of traveling to a given drug market and other parts of the metropolis. In turn, such travel may lead to encounters with other PWID, and the establishment of drug injection networks. There are several drug markets in well-defined geographic areas within metropolitan Chicago (Fig 2) [39]. When two PWID access the same market, they are relatively more likely to establish a connection as compared to PWIDs who do not (See S1 Text, Eq 1).

**Table 4:** Detailed statistics of the PWID population in APK. The initial 2010 PWID population is gradually removed through attrition, and is replaced by the population of newly-initiating PWID. Values for the newly-initiating population reflect attributes at the time of joining the PWID population. Ages and drug-related risk behaviors reported are mean values. All values for newly-initiating PWID are significantly more likely to be young and NH White.

	PWID Jan. 2010	PWID newly initiating-2010–2020†	PWID Dec. 2020	Combined PWID population: Initial (2010) and newly-initiating (2010–2020)						
				North Side	South Side	Suburban	Under 30	Over 30	HR	Non-HR
**Total**	32000 (±1080)	8499	32000 (±1090)	4032	6134	23103	19178	21334	19603	20909
**Female (%)**	30 (±4)	36	32(±9)	27	34	32	35	28	31	31
**Male (%)**	70(±4)	64	68(±9)	73	66	68	65	72	69	69
**NH Black (%)**	21(±1)	11	17(±6)	11	68	3.6	3.0	32	24	13
**Hispanic (%)**	19(±6)	18	19(±6)	22	12	11	15	22	22	15
**NH White (%)**	57(±2)	67	60(±2)	60	19	81	78	43	51	68
**Age (years)**	35(±0.6)	26	40(±2)	34	42	29	24	42	34	33
**Elapsed years of injection drug use**	11(±1)	0.1	16(±2)	10	17	6.0	3.2	14	9.4	8.6
**Daily injections**	2.5(±0.04)	2.4	2.5(±0.04)	2.4	2.7	2.3	2.4	2.5	2.7	2.2
**Fraction receptive sharing** **	0.19(±0.03)	0.20	0.19(±0.03)	0.12	0.14	0.25	0.21	0.19	0.024	0.36
**In degree** *	0.51(±0.03)	0.59	0.53(±0.03)	0.43	0.42	0.57	0.60	0.43	0.37	0.64
**Out degree** *	0.45(±0.03)	0.48	0.46(±0.03)	0.40	0.40	0.50	0.54	0.38	0.33	0.57
**HR (%)**	49(±3)	49	50(±4)	68	66	34	48	49	100	0.0
**Non-HR (%)**	51(±3)	51	50(±4)	32	34	66	52	51	0.0	100
**HCV RNA+ (%)**	29(±3)	5.1	26(±4)	22	42	20	14	35	23	26
**HCV antibody + but not infected (%)**	14(±3)	0.0	10(±2)	8.2	17	7.9	5.0	15	10	10

* The “in-degree” is the number of the in-network relationships, i.e. the number of persons from whom the PWID receives drugs or other risks, while the “out-degree” is the number of connections to which the PWID gives drugs or other risks.

**Fraction of injections involving receiving drugs or injection equipment from another person in the network.

^†^Variability data is not available. HR = PWID in Harm Reduction programs (e.g. needle exchange programs). nonHR = PWID not in harm reduction programs

### Summary of simulated processes

The simulation uses the discrete event simulation (DES) methodology [53], whereby the simulation steps from event to event. Many types of events take place on a regular daily basis, particularly drug injection, sharing, and HCV transmission. PWID attrition, cessation or dissolution of relationships occur infrequently based on a stochastic schedule.

We applied the following steps to introduce PWID into the simulation:

1. An individual X is drawn at random from the CNEP+ database. X already contains most of the attributes (Table 3) of a PWID: race, gender, age, network size, HCV antibody test result, etc.
2. All of the attributes of X are copied to the synthetic PWID Y, including specific ID number, race, gender, location (Table 3).
3. The HCV infection status of Y (acute/chronic/recovered) is assigned stochastically based on the HCV antibody test result attribute assigned within the CNEP+ database (see Table 2).
4. Y is added to the PWID population and begins his/her drug career. Network connections are established as described above.

During the course of the simulation, each PWID performs the following actions:

1. Daily drug injection and possibly needle receiving/giving (with frequency specific to the individual, including potential HCV infection).
2. Update of HCV infection stage for PWID going through 1^st^ acute phase or acute phase upon re-infection.
3. Update of both the IDU biological age and his/her injection drug career age.

In addition, APK carries out the following processes:

1. Formation of relationships between two PWID (only for PWID who maintain a network and have recently lost a relationship).
2. Removal of PWID from the population, due to mortality, incarceration or cessation of drug use.

At the beginning of every simulated day, the simulation calculates the number of injections each PWID would perform, which may be zero, one, two and so forth. PWID are then selected stochastically to perform the injections. If a PWID has a non-zero probability p of receptive sharing, then an average fraction p of the injections would involve selecting a partner from the PWID’s in-network.

### Software modeling platform and tools

We developed APK based on the Repast Symphony 2.1 open source platform, which was created by Argonne National Laboratory (Argonne, IL) [53,54]. We built APK in the Java 7 programming language that allows for simulation of hundreds of thousands of individuals and we optimized and streamlined it to accommodate large geographical areas and populations. Each simulation uses a single computer core, and runs for 11 simulated years including 1 year of burn-in, except for one simulation noted below which used 21 years including 1 year of burn-in. We wrote all data analysis tools using the open source Python 2.7 programming language.

### Statistical and sensitivity analysis procedures

We performed sensitivity analysis procedures where the values of the parameters were changed and the simulation repeated 300 times. We performed simulations using the Stampede supercomputer at the University of Texas at Austin and the Elastic Compute Cloud from Amazon Web Services. Because of the high computational cost of each simulation (~1 hr), our sensitivity analysis procedure used Latin Hypercube Procedure to maximize coverage of the parameter space [55] and to accelerate the data collection substantially. Parameter values were drawn from a normal distribution with the indicated mean value and truncated at the indicated value. For each setting of the parameter values, we initialized the PWID population and simulated the population and recorded the HCV epidemic. Incidence rates were calculated by counting acute HCV infections, and for each PWID who became infected, tracking the time from entering the simulation to the time of infection.

## Results

### Validation of APK

#### Software Verification

To augment the accuracy of the APK program, two programmers (A.G. and A.H) performed a verification procedure [56] that includes code walkthroughs, profiling and parameter testing to ensure the model was working as intended. These tests reduced the risk of error in the programming of the model. The verification procedure was complemented by validation of the model with empirical data.

#### Validation of HCV prevalence predictions

**Fig 4:**
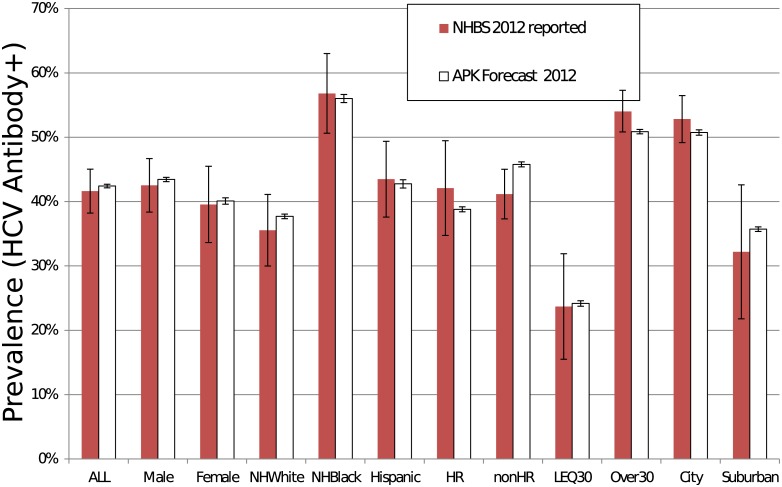
Comparison of APK predictions for 2012 with the NHBS empirical survey data from 2012. APK correctly forecasts the prevalence overall and in 11 of 11 subgroups with substantially different prevalence values (average error in estimate: 2.0%). Error bars represent one standard deviation. NHWhite = Non-Hispanic White; HR = Individuals in Harm Reduction Programs; nonHR = Individuals not in Harm Reduction Programs; LEQ30 = PWID aged 30 or younger; Over30 = PWID over 30 years of age; City = PWID within the City of Chicago; Suburban = PWID living in Chicago suburbs.

We validated APK using the 2012 NHBS survey, the most recent representative data available on PWID for metropolitan Chicago. We utilized the 2012 NHBS survey to construct a synthetic “validation” population for 2012. To do this, we applied the same imputation procedure as APK (i.e. both based on CNEP+) to generate the population characteristics, then applied the 2012 NHBS HCV prevalence. The resulting validation population was then compared to the HCV prevalence predicted from the APK simulation initiated in 2010. The validation results show high concordance, i.e., the predicted and actual values match within 2% overall and one standard error in 11 out of 11 subpopulations (Fig 4).

#### Network structure validation

**Fig 5:**
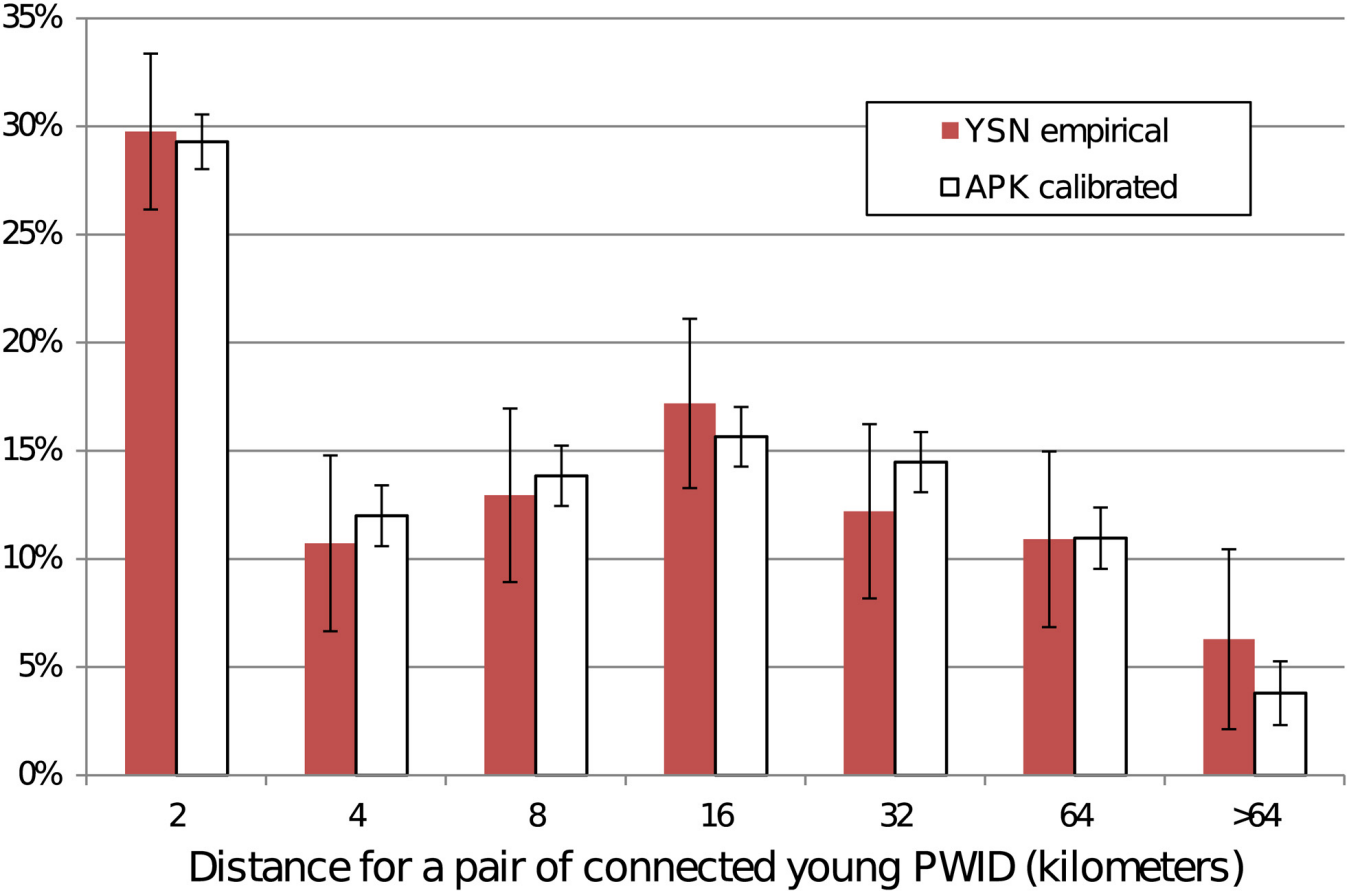
Distances in the drug-sharing network of among young PWID (age 30 or younger). The data shows the results for APK compared to empirical data from the Young Social Network (YSN) dataset. Error bars represent one standard deviation.

We used raw data obtained from a social network study among young PWID (from the Young Social Network Study) to calibrate and validate the network formation process by comparing the distances between pairs of PWID that exchange drugs (Fig 5). The simulated and actual networks match closely with an average error of 1.3%. For example, in about 30% of the pairs, the two PWID are geographically located within 2 km of each other for both the empirical data and APK networks. The likelihood drops significantly within the empirical data beyond 2 km, but has a second peak at 8–16 km; this drop beyond 2 km and the second peak are reproduced in APK.

### Composition of the PWID population is expected to change through 2020

**Fig 6:**
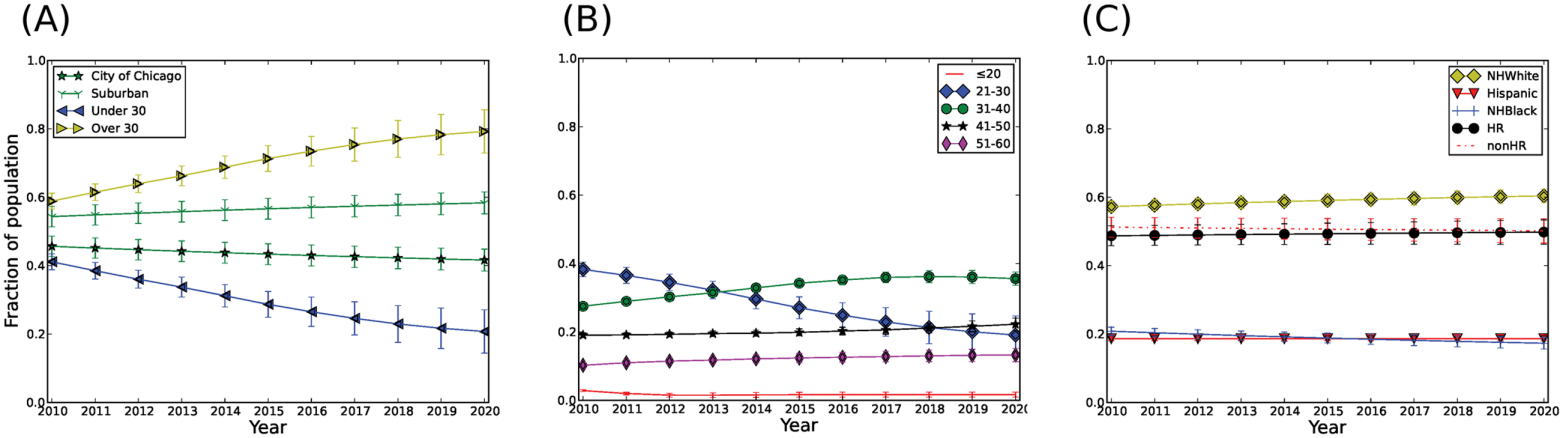
Age and Composition of the PWID population from 2010–2020. (A) Composition of PWID from 2010–2020 based on location, Suburban and City, and age, persons over and under 30 years of age. (B) Detailed distribution of PWID over time within different age groups <20; 21–30; 31–40; 41–50 and 51–60 years of age, (C) Composition of PWID population within racial groups (NH Black, Hispanic and NH White) and within HR and non-HR groups, In all figures trends show average of 300 simulations and errors represent one standard deviation between simulations. HR = PWID in harm reduction programs, non-HR = PWID not enrolled in harm reduction programs.

The composition of the population was studied over the period from January 1, 2010 through December 31^st^ 2020. The simulation indicates some significant changes in the Chicago PWID population by 2020. First, the proportion of the PWID population from suburban compared to urban Chicago areas is forecast to remain unchanged (or slightly increase) from 54% (SD: 3%) in 2010 to 58% (SD: ±3%) in 2020 (Fig 6A). Second, due to the aging of existing PWID and relatively low rates of attrition and long injection drug use careers (i.e. low cessation), the proportion of PWID >30 years old is predicted to increase substantially over time with a corresponding decrease in the proportion of those <30 years old (Fig 6A), resulting in an average age increase from 35 yr in 2010 to 40 (±1.8) yr in 2020 (data not shown). Specifically, young PWID aged 21–30 are predicted to decline as a fraction of the population from 38% in 2010 to 22% (±6%) in 2020 while the fraction of PWID in their 30s, 40s and 50s would increase (Fig 6B), with the greatest increase occurring in the 31–40 age group. Third, the PWID population is projected to become increasingly non-Hispanic (NH) White (increasing by 3% to 60±2%) and less NH Black (decreasing by 3% to 17±2%) (Fig 6C). However, the proportion of PWID engaging in harm reduction (HR) practices is expected to remain steady, changing from 49% (SD: ±3%) in 2010 to 50% (SD: ±4%) in 2020 (Fig 6C).

As expected, there is significant regional variation in racial/ethnic composition and HCV prevalence between 2010 and 2020 with the South Side of Chicago being predominantly NH Black (68%) with high anti-HCV prevalence (17%), while the suburban areas are predominantly NH White (81%) with lower anti-HCV prevalence (8%) (Table 4). Compared to the initial 2010 PWID population, newly-initiating PWID (2010–2020) were more likely to be NH White (67% vs. 57% in the initial 2010 population) and less likely to be chronically infected (i.e., HCV RNA positive) (5% vs. 30% in the initial 2010 population) (Table 4).

### Network structure of the PWID population

The network structure of the PWID population at the beginning of the simulation (2010) is characterized by the presence of many isolated individuals, with the 32,000 PWID connected by just 14,300 directed connections. These connections establish approximately 1300 strongly connected components of more than one person, i.e. sub-networks with reciprocal exchange of drugs, the largest of which has size 45 (for terminology see [57]). In 2010, a majority of PWID (68%) do not receive drugs or equipment at all (in degree parameter, Table 4), and consequently are not at risk for injection-related HCV infection.

Networks in APK reflect community areas with high homophily on select demographic characteristics (e.g. race/ethnicity). For example, NH Whites in select urban and most suburban areas are more likely to live near other NH Whites, which is expected to result in an increased probability of encounter and formation of drug partnerships among NH Whites in the APK model. In addition, racial/ethnic network homophily among PWID is documented in epidemiological studies [1,39]. This pattern is realized in APK, which showed more racially homophilic connections than would be expected if the mixing were random, i.e. the majority of NH Blacks and NH Whites are connected to others of the same race in the APK model (Table 5). Consistent with a recent study on young Chicago PWID (Boodram personal communication), NH Whites showed higher racial/ethnic homophily with their injection network members compared to Hispanics (all races) (80% vs 41%).

**Table 5:** Statistics of actual network connections in APK by race/ethnicity. NH Whites and NH Blacks have high racial/ethnic homophily with injection networks; therefore, HCV incidence in NH Blacks and NH Whites is driven by background prevalence in respective ethnic/racial groups.

	To:	NHWhite	Hispanic	NHBlack	Other	* *Total*
**From**:	**NHWhite**	80%	12%	5%	3%	58%
	**Hispanic**	43%	41%	12%	4%	18%
	**NHBlack**	28%	21%	50%	2%	21%
	**Other**	66%	18%	7%	9%	3%

*Total gives the fraction of the PWID population in that demographic group in 2010, and is the expected number of relationships under random relationship formation.

### Prevalence of HCV will decline by 2020

**Fig 7:**
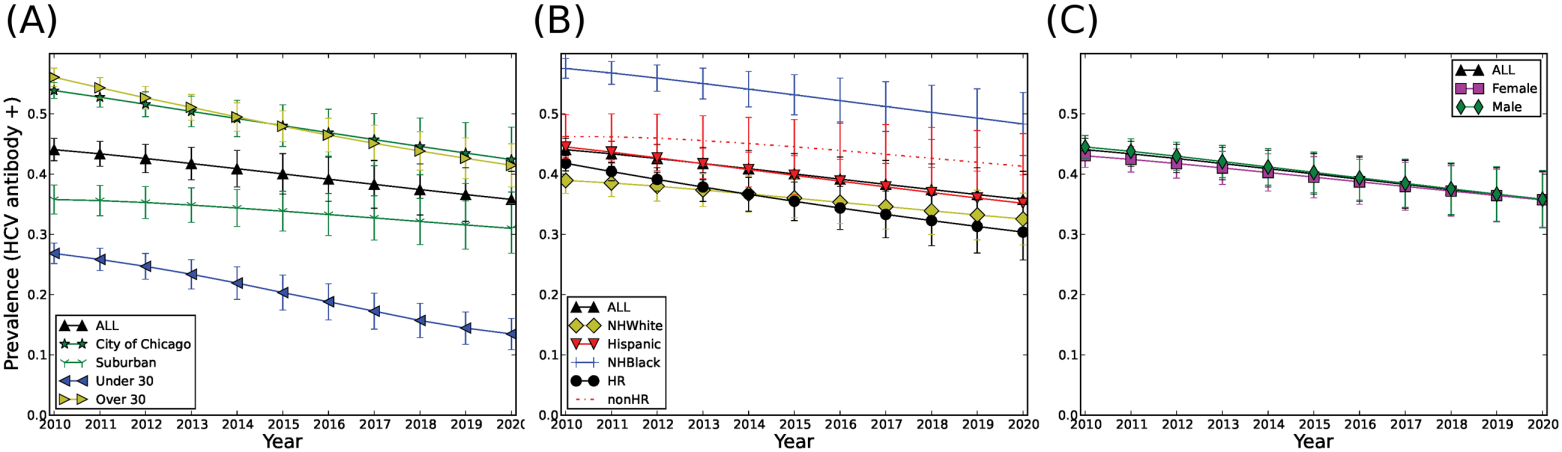
Forecast of HCV antibody prevalence in Chicago over a 10-year span, 2010–2020. (A) HCV antibody prevalence based on location, Suburban and City, and age, persons over and under 30 years of age. (B) Prevalence within the total population, individual racial groups (NH White; NH Black and Hispanic) and HR and non-HR groups. (C) Prevalence within the total population and based on gender. Trends show average of 300 simulations and error bars represent one standard deviation. HR = PWID in harm reduction programs, non-HR = PWID not enrolled in harm reduction programs.

The APK forecast for HCV prevalence, as measured by HCV antibody positivity, in metropolitan Chicago is reported in Fig 7 and is expected to decline overall at a rate of 0.4% per year from 43% in 2010 to 39% in 2020 (Fig 7A). However, the rate of decline from 2010–2020 differs by age, racial/ethnicity, and harm reduction group designation. For instance, HCV prevalence among PWID ≤ 30 years old is forecast to decline more precipitously (27% to 13%) compared to those >30 years of age (56% to 41%) (Fig 7A). By race/ethnicity, HCV prevalence decline is expected to be greater among Hispanics (all races) (from 44% to 35%, ±5%) and among NH Blacks (from 57% to 48%, ±5%) compared to NH Whites (from 39% to 33%, ±4%) (Fig 7B). As expected, those enrolled in needle exchange/harm reduction programs would experience a steeper decline in HCV prevalence (from 42% to 30%, ±5%) compared to those who are not (from 46% to 41%, ±5%) (Fig 7B). By gender, HCV prevalence decline is forecast to occur at similar rates among females (from 43% to 36%, ±5%) and males (from 44% to 36%, ±5%) (Fig 7C).

Although long term projection is associated with more uncertainty, an extended simulation for years 2020–2030 showed that anti-HCV prevalence would continue to decline from 39% to 33% between 2020 and 2030 (data not shown). However, due to expected higher incidence among the younger population over time (Fig 7), we project that HCV prevalence among the younger population will not decline as steeply and will plateau around 13% in 2030 (data not shown).

### Incidence of HCV varies by sub-group

**Fig 8:**
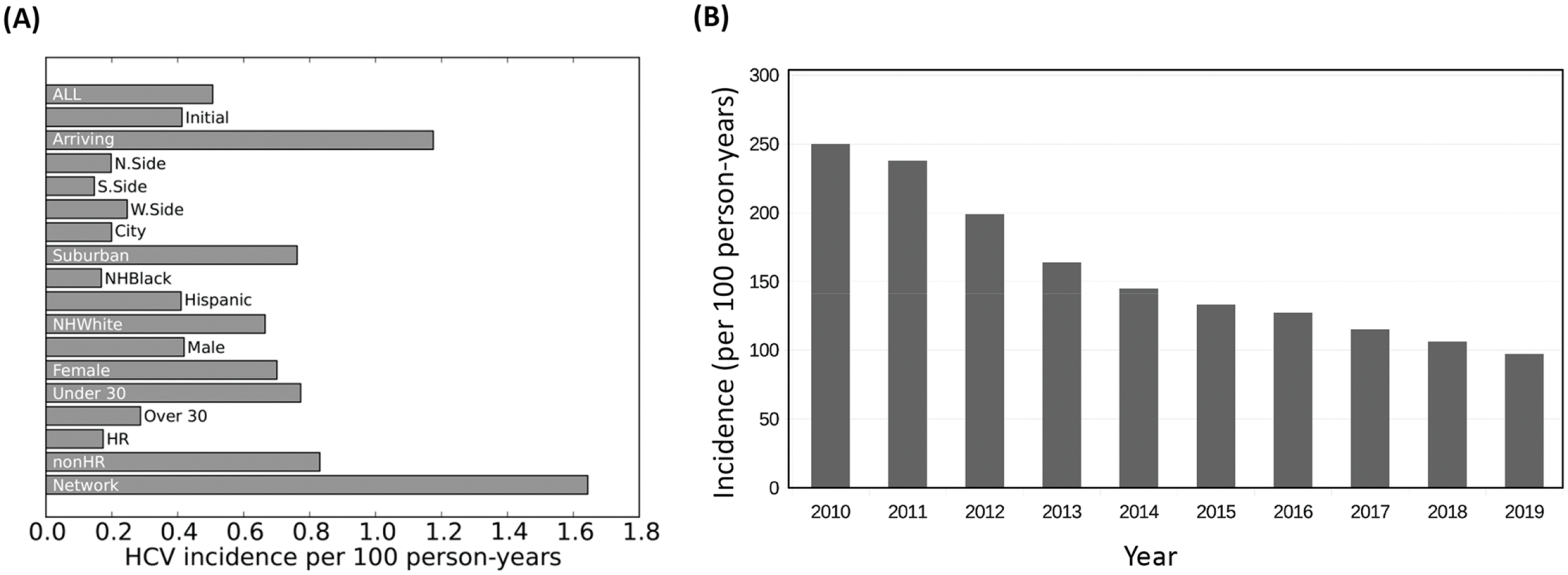
Incidence of HCV among PWID in metropolitan Chicago. (A) Incidence density by group and geographic area summed over 2010–2019. (B) Total incidence of HCV by year. Values are expressed as HCV incidence per 100 PY and have an estimated uncertainty of ±20%. HR = Individuals in Harm Reduction Programs; nonHR = Individuals not in Harm Reduction Programs. Network = Individuals having at least one incoming connections in the PWID network.

Fig 8A shows the HCV incidence per 100 person-years (PY) among PWID from 2010–2020. HCV incidence overall is estimated at 0.51 per 100 PY, with significant differences between sub-populations. The primary sub-groups driving incidence are those with an injection network, those arriving into the population after 2010, and suburban PWID. NH Whites are more likely to be members of all three of these sub-groups between 2010–2020, which would account for a three-fold higher incidence in this group compared to NH Blacks and Hispanics (0.66 vs. 0.17 and 0.41 per 100 PY, respectively). The HCV incidence among all PWID with at least one in-network injection partner is 1.2 per 100 PY, with an even higher incidence among this group if they are also young and newly arriving into the population (3.3 per 100 PY) (data not shown). Compared to their respective counterparts, those enrolled in harm-reduction programs, older PWID >30 years, and male PWID have lower HCV incidence (Fig 8A). Analysis of the incidence over time for all populations (Fig 8B) shows that incidence declines from approximately 250 cases per year in 2010 to under 100 by 2019. This is consistent with a rapidly declining prevalence we reported in Fig 7.

### Rapid HCV Acquisition within the first years of injection career

**Fig 9:**
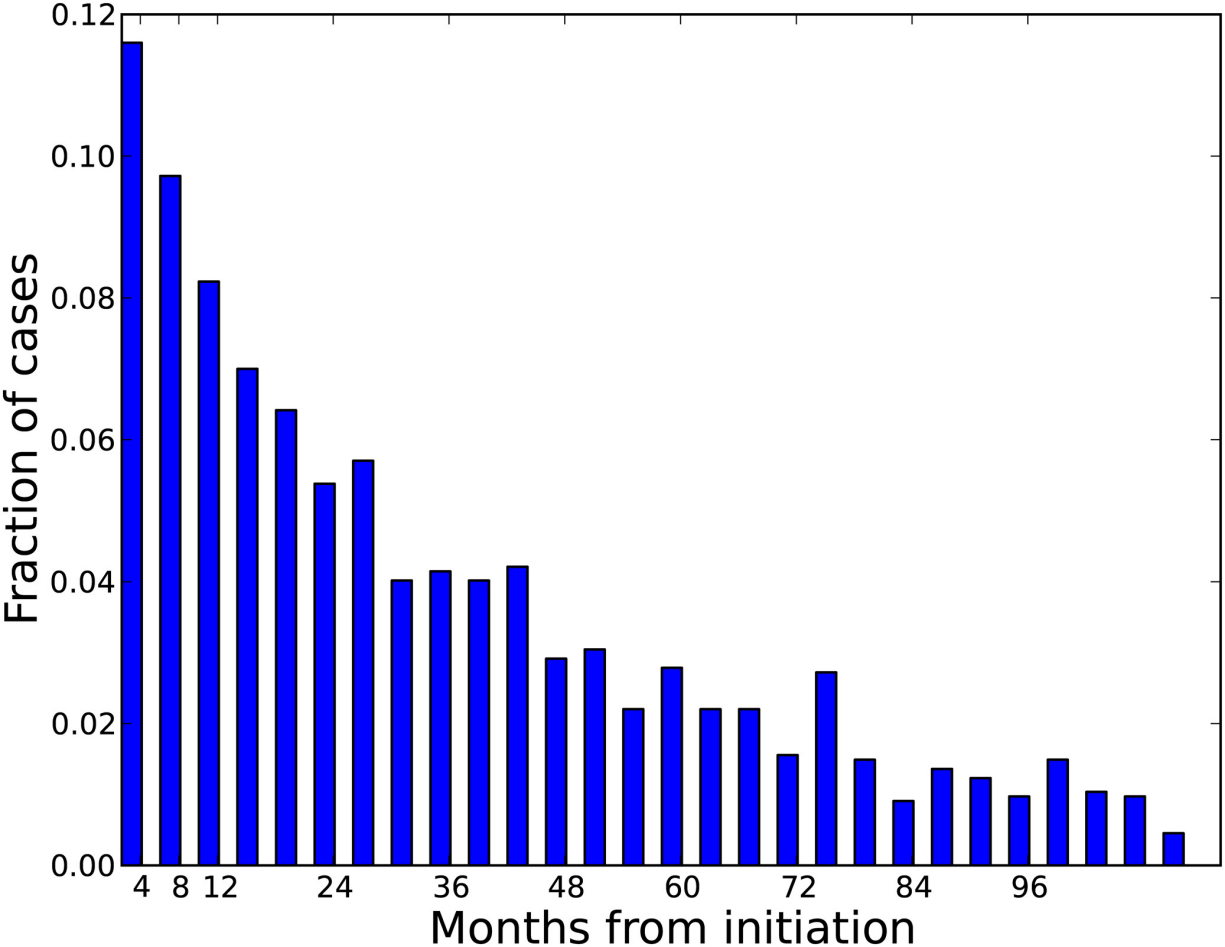
Timing of HCV infections over the duration of the injection career, among PWID who become infected. The horizontal axis indicates months from the beginning of injecting drug use. Each bar represents a 4 month period.

**Fig 10:**
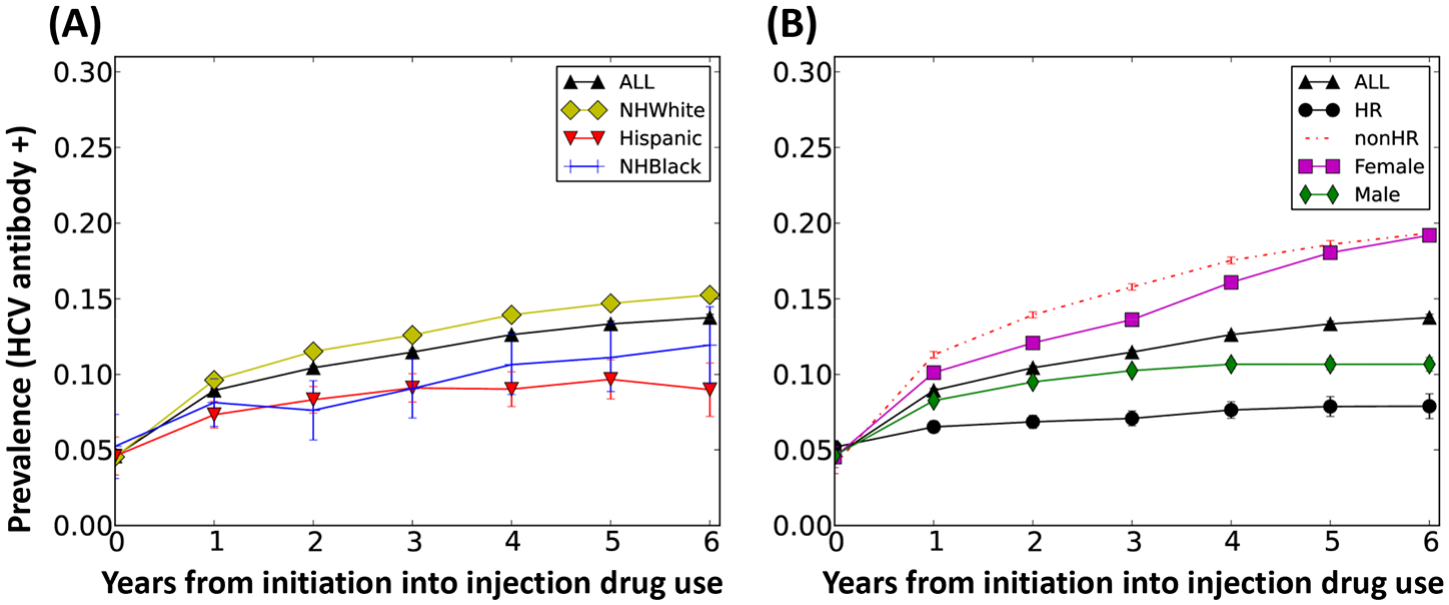
The prevalence of HCV over the injecting career among PWID who initiate into injection drug use. (A) Prevalence within the total population and within individual racial/ethnic groups, NH White, Hispanic, NH Black. (B) Prevalence within the total population and divided for gender and harm reduction (HR) enrollment. Time is counted from the beginning of an individual’s initiation into stable injection drug use (and APK), and assumes 5% incidence due to experimental use of Heroin before transition to stable injection use. The curves represent the combined injection careers of all PWID who initiated over 2010–2020. Data is shown as the mean of 300 simulations and the error bars represent one standard deviation.

We tracked the injection career length for all PWID in the simulation, which we then used to estimate the timing of new HCV infections. An estimated 29% of HCV infections are expected to occur during the first year of injecting drug use and another 18% during the following year (Fig 9). The probability of HCV acquisition slowly declines during subsequent years due to infection saturation of the population. This pattern is also reflected in the higher incidence found among new initiates into injection drug use (i.e. arriving sub-group in Fig 8A), which ultimately also results in a sharp increase in anti-HCV prevalence among this group by the end of the first year of injection drug use that stabilizes over the subsequent years (Fig 10A). Among newly-arriving PWID during 2010–2020, it is notable that NH Blacks are expected to experience lower HCV prevalence over time as compared to NH Whites. This may reflect both changes in high risk behaviors and network composition over time. We observed a sharper increase in HCV prevalence in females initiating into drug use than males (Fig 10B), while the lowest increase in prevalence over the first year is seen in newly initiating PWID enrolled in harm reduction programs (Fig 10B).

## Discussion

Our study describes a novel agent-based model (termed APK) for the PWID population in metropolitan Chicago and applied this to study and predict changes in HCV prevalence in this population. A barrier to developing realistic prediction models for PWID is a lack of empirical-grounded data that includes biological, behavioral, network and geographical parameters. We addressed this shortcoming with APK by combining multiple diverse large-scale datasets (Fig 1) derived from empirical studies on metropolitan Chicago PWID to develop a representative PWID population. In APK, each individual PWID is based on a profile developed from these empirical data that includes demographic characteristics, risk behaviors, HCV infection status, place of residence, and injection network size. The metropolitan Chicago geography is represented in APK by zones based on the 2010 US Census ZIP code level data. Geographical distance between the zones is considered in the probability of network connections among PWID and is also critical for forecasting HCV at the neighborhood level and among the suburban PWID. In addition to these internal features, APK includes sensitivity analysis capabilities and an advanced graphical user interface that enables exploration of a running simulation and visualization of the geographic and demographic distribution of the disease.

Using APK, we investigated the long-term trends for the PWID population in metropolitan Chicago and HCV infections among PWID. Overall, the model predicts that the PWID population would increase in mean age substantially by 2020 (Fig 6), while the overall prevalence of HCV would decline (Fig 7). As expected, PWID enrolled in harm reduction programs (HR) are forecast to experience a sharper decline in prevalence (1.1% per year) compared to those not enrolled in harm reduction programs (nonHR) (0.5% per year) (Fig 7B). This finding may reflect the underlying effectiveness of harm reduction programs in preventing HCV infection, as well as the overall smaller networks and lower frequency of needle sharing in this population compared to non-harm reduction PWID. Among racial/ethnic groups, we project that NH Blacks will experience a relatively rapid decline in HCV prevalence (Fig 7B), but still continue to have the highest prevalence overall. This pattern is similar to the harm reduction group; as such, compared to NH Whites; NH Blacks have smaller injection networks (average in-degree 0.61 vs. 0.41, respectively) and lower frequency of needle sharing (0.14 vs. 0.22 per injection episode). These differences in network size and frequency of needle sharing in the NH White population could account for the lower rate of prevalence decline in this subgroup (Fig 7B).

We project the overall HCV incidence rate to be 0.51 per 100 PY, which is low in comparison to empirical studies from large cities such as Baltimore and Seattle (47 and 9.8 per 100 PY, respectively) [58]. However, Chicago is notable for relatively low incidence; the most recent estimate of HCV incidence among Chicago PWID comes from the CIDUS III study (2002–2004) [24,59], which reported incidence of 6.0 per 100 person-years and is based on only a 6-month follow-up of 18–30 year olds with relatively short median injection career length [24,59,60]. In contrast, APK encompasses wider geographic and racial/ethnic diversity among the population in APK. Nonetheless, this decline in incidence has been observed in other cities (e.g. Vancouver, Canada [61]) and might be found elsewhere but be underreported because some subgroups show relatively stable trends, as shown in Fig 7. HCV incidence is heightened among the newly-initiating sub-population of PWID (Fig 8A) who are also more likely to be younger (<30 years) and suburban rather than longer-term, older PWID (Table 4). For example, young newly-initiating PWID with at least one network relationship have an HCV incidence in APK of 3.3 per 100 PY, while all PWID over 30 years report an HCV incidence of 0.3 per 100 PY (Fig 8A). For these reasons, we expect that by 2020 the epidemic will be increasingly found among suburban NH White PWID who are not enrolled in harm reduction programs. These predictions from the APK model are consistent with previous studies showing an ongoing shift in the racial composition of PWID populations throughout the United States [8–10,62,63]. Previous studies also showed that the majority of new HCV infections occur during the first or second year of injection drug use [25,64] and the incidence is heightened among females as compared to males [65], which agrees with our findings (Figs 9 and 10B).

Populations at high risk for acquiring HCV may be suitable candidates for future vaccine clinical trials. Although a number of promising vaccines are being developed [66,67], testing of those vaccines is complicated because of the complexity of the PWID population [68]. We expect that populations in which we predict elevated incidence of HCV would be the best candidates for testing possible vaccines. Based on our findings, such studies might preferentially recruit from PWID <30 years old not enrolled in harm reduction programs. Although this may be a challenging group to target our findings indicate that the development of novel recruitment methods that are designed for this population, such as mobile apps, should be vigorously explored and would be highly beneficial in the long-term.

We found that the overall prevalence of HCV is expected to decline, which is consistent with the attrition process in the PWID population, and is not unexpected when viewed in a historical context. The APK-predicted rate of overall decline (8% over 10 years) is within historical trends for HCV among Chicago area PWID. For example, according to the three CIDUS studies that recruited both harm reduction and non-harm reduction PWID, the prevalence among Chicago PWID 18–40 years old declined from 70% (CIDUS I, 1994–96) to 27% (CIDUS II, 1997–99), and then to 14% (CIDUS III 2002–04) [16]. Our long term forecast for 2020–2030 is for prevalence to continue to decline but at a slower pace.

A number of limitations and simplifications inherent to APK must be considered when interpreting our findings. First, to generate the synthetic population, APK relies on data from the COIP Needle Exchange Program and, although some corrections were applied, our representation is less accurate of PWID who are not enrolled in the needle exchange program (non-harm reduction) or who may live too far to access COIP’s services. Second, our network construction parameters are fitted to young social networks data that includes only PWID 30 years old or younger, but older PWID might have different network structures. A better understanding of the non-harm reduction and older populations would increase the accuracy of the simulations and this component of APK will be refined in future when these data become available. Third, while APK considers aging and the duration of the drug career, it does not model how age affects drug behaviors and risk awareness. We examined this assumption using cross-sectional data from the CIDUS III study [59] and confirmed that injection behaviors, including network size, number of daily injections and frequency of sharing show no consistent association for about ten years of drug use. Indeed, we saw that APK can accurately forecast HCV prevalence three years into the future, suggesting that behavioral changes by individual PWID do not play a significant role in the spread of HCV in the population as a whole. Fourth, APK simplifies the interrelated processes of attrition and cessation of injection drug use. In particular, APK uses an exponential model of incarceration and mortality, and extrapolates the duration of injection drug careers based on data from the Los Angeles, CA area [48]. Only long-term follow up studies (>30 years) of PWID in the Chicago area would provide the necessary data to validate this aspect of the simulation. Fifth, APK accounts for place of residence and the drug markets but does not directly model daily mobility of the PWID. Places of work, pharmacies, hospitals, and other sites could be also important for the transmission of HCV, however their epidemiological role, unlike airborne pathogens [69] is expected to be secondary to the place of residence and the drug markets for HCV spread. Similarly, although APK considers the formation of relationships through encounters, other processes might play a contributing role in relationship formation, such as introduction via mutual friends.

Despite those concerns, we suggest that our findings are robust to these uncertainties, as demonstrated by validation with empirical data (Figs 4 and 5). The majority of the uncertainty is accounted for by the sensitivity analysis procedure and shown in Figs 6 and 7. Additionally, based on our validation studies, we believe that the simulation can provide accurate forecasting for three years and as such the best application for our simulations would be to evaluate scenarios such as public health interventions. The forecasting range APK provides is comparable to the duration of medium-term epidemiological studies, such as those that consider syringe exchanges, behavioral and medical interventions.

Overall, APK is the first detailed and data-rich agent-based model available for the PWID population in metropolitan Chicago and may be the most detailed model worldwide [17]. It could be adapted to other cities in the US and worldwide based on a comprehensive survey of the PWID population including their geographic locations, network connectivity and HCV infection status. APK could also consider infections (including co-infections) with hepatitis B or HIV and to evaluate intervention strategies such as anti-HCV antiviral treatment scale up and HCV vaccine trial design and evaluation.

## Supporting Information

S1 Text Supplemental Methods. (DOCX)
