# Supplementary material for: Agent-Based Model Forecasts Aging of the Population of People Who Inject Drugs in Metropolitan Chicago and Changing Prevalence of Hepatitis C Infections
Source: PLoS One. 2015 Sep 30;10(9):e0137993. doi: 10.1371/journal.pone.0137993 (PMC4589282; doi:10.1371/journal.pone.0137993)
Supplement: S1 Text — (DOCX) [file pone.0137993.s001.docx]

# Agent-based model forecasts aging of the population of people who inject drugs in metropolitan Chicago and changing prevalence of hepatitis C infections

**Supplemental Methods**

**CNEP+ Population**

The PWID population in metropolitan Chicago was divided into two classes: (1) those that receive harm-reduction interventions (HR), and (2) those that do not (non-HR). It has been reported ([1](#_ENREF_1), [2](#_ENREF_2)) that an estimated 69% of the young Chicago PWID obtains needles from safe sources. Although all COIP services are within Chicago city limits, the suburban population is highly represented in COIP; 38% of the CNEP users reside outside the city limits and travel to the city for COIP services.

Previous studies in Chicago found that the non-HR population and the CNEP enrollees have no statistically significant differences with respect to gender, race/ethnicity, marital status, education, sexual orientation, main source of income, and other attributes ([3](#_ENREF_3)), but differ in age and several drug-related behaviors. Based on this information, we constructed the Enhanced CNEP (“CNEP+”) dataset that contains CNEP as well as the profiles of Chicago-area PWID not enrolled in CNEP. CNEP+ is designed as a synthetic yet large and unbiased sample of the actual PWID population, and CNEP+ is the set from which the simulation constructs new PWID by copying their attributes (including demographics, place of residence, health and behavior). CNEP+ contains copies of all the CNEP responses, and they represent the HR class. CNEP+ also contains profiles that represent non-HR individuals. Because HR and non-HR are demographically similar ([3](#_ENREF_3)), non-HR profiles were constructed (1) by copying the demographic information of CNEP profiles; (2) the copies were adjusted for differences between HR and non-HR population ([3](#_ENREF_3)) to match the survey-based frequency of risky behaviors and health (see main text, Generation of the Synthetic Population).

Table 1 shows the parameters used for constructing the CNEP+ population. In step (1) (**mean_enrichment_Chicago** and **mean_enrichment_suburbs**), the number of copies varies between 0.5 and 2 depending on the enrollment rate of the needle exchange as a fraction of the total PWID population in the geographic zone. In step (2), adjustments were made in the age (**nonHR_age_boost)**, drug career duration **(nonHR_age_started_boost)**, in and out network size **(nonHR_degree_boost) (**the **in-network** partners are people giving drugs to the PWID, while the **out-network** are those receiving drugs from the PWID), HCV antibody positivity **(nonHR_hcv_ab_boost)**, number of injections (**nonHR_injection_ratio)** and frequency of receptive sharing (**nonHR_sharing_boost)**. Generally, in Chicago and unlike in certain other cities, the non-HR population exhibits greater propensity for risky behaviors ([2](#_ENREF_2)).

A total of 6006 valid rows are available in the original CNEP, of which 3694 are in Chicago and 2309 are in the suburban communities. The non-HR profiles include 1900 profiles in Chicago and 4750 profiles outside Chicago. Thus, a total of 12400 profiles represent the metropolitan PWID population of approximately 32000 ([4](#_ENREF_4)).

**Network component**

***PWID encounter rates***: Network connections arise only when two PWID encounter each other. Therefore, a very central calculation for network formation is to determine the probability that two persons are likely to encounter each other and form a drug relationship. APK allows for three kinds of encounters (1) in the neighborhood close to the place of residence; (2) at a drug purchase market, and (3) other encounters, which includes co-workers or any stranger in the city. The following formula gives the total encounter rate between PWID at any pair of ZIP codes i and j at geographic distance :

**Equation S1.** The rate of encounters between persons in zones i and j.

where and are the available population in zone i and zone j, respectively.

The parameter values are given in Table 2.

**interaction_home_cutoff (****):** the mean size of the PWID’s neighborhood. The value of might be smaller or larger than a typical ZIP code zone. If it is smaller, it still includes everybody in the ZIP code; if it is larger, it might include one or more additional ZIP codes, if their geographical center is within kilometers measured by a straight line from the center of the “home” ZIP code.

**interaction_rate_constant** (): the rate of encounter for a pair of PWID who reside in the same home zip code zone.

**interaction_rate_at_drug_sites** (): the rate of encounter at drug markets. Finally, **interaction_rate_exzone** (): the rate parameter for encounters due to all activities (work, shopping, etc except activities related to the drug market) for people who do not live in the same neighborhood.

The above parameter values were calibrated based on YSN network data, by comparing the young PWID in the YSN study, to young PWID in APK. For calibration we used an iterative error minimization procedure in which the parameters were set to an initial value, and the resulting network was compared to the network in YSN, in terms of the geographic distances in the relationships. The parameters were then repeatedly adjusted until no further improvement appeared to be possible, after 20 iterations.

The formation of network connections follows these specific steps:

1. The simulation computes the encounter rate between all the ZIP code zones, based on factors such as the distances between them (see Equation S1).
2. Every day, the software examines the networks of all the PWID as if in a census. For each ZIP code, all PWID that have fewer connections than their individual target value are added to a list of “available” PWID. This allows us to remove from consideration PWID which would not form new connections.
3. Encounters between PWID located in every pair of zones i and j are planned ten times each day. For each pair of zones i and j, APK computes a waiting time until the next encounter event. The waiting time until the encounter is based on the exponential distribution with a rate from Equation 1, and the events are scheduled sequentially based on their waiting time. The higher the encounter rate between a pair of zones, the more events are scheduled.
4. At each encounter event, a random pair (x,y) of PWID from the list of “availables” is selected (x is from zone i, and y is from zone j), and a connection is attempted (details below).
5. The scheduling of encounters continues until 24 hours have elapsed. The number of new relationships is several thousand per day when the simulation is initialized, and falls to about 20 after the network achieves a dynamic equilibrium.

At the initialization of the simulation, APK attempts to ensure that most PWID have the correct number of connections, i.e. network size. Briefly, APK computes the total number of receptive relationships that should exist in the PWID network by summing the in-degree attribute of all of the PWIDs. An identical sum is computed by summing the out-degree of all the PWID. Now APK initiates a process of repeated census followed by linking (steps 2-5 above). After completing step 5, APK compares the total number of links to the pre-computed sums and ends the linking process when 95% or more of the network is complete, i.e., when the actual number of receptive and giving links are 95% of the total in-degree and out-degree, respectively, whichever occurs first. To prevent excessive work, up to a maximum of 30 consecutive rounds are attempted. Generally, because the in and out sizes of the nodes are statistically different, it is impossible to construct a network where all the PWID would have the target number of in and out connections. About 90 to 95% of the possible links are successfully established: there are approximately 13600 connections whereas the total maximal in-network and out-network sizes are 16500 and 14500, respectively. Notice that because the two numbers differ, at most 14500 are possible in the network.

***Formation of network connections at encounters:*** At any encounter event, two PWID X and Y have an opportunity to form a relationship, which may lead to sharing of drugs or injection equipment. Only some encounters produce a relationship because it is well known that people tend to connect with those who have similar backgrounds, generational groups and other commonalities. This idea is called homophily ([5](#_ENREF_5)) and to represent homophily the simulation computes the demographic distance (DD) between X and Y before a connection is established. The demographic distance DD(x,y) consists of 2 factors which are summed and divided by 2: (1) absolute age difference divided by 10, (2) difference in race (0 if same, otherwise 1).

In total, an encounter produces a connection in the following way:

1. When X encounters Y, a connection may or may not be formed.
2. X must have fewer connections that his or her pre-set maximum out-network size; similarly for Y’s in-network.
3. A connection preferentially succeeds if X and Y are demographically similar to each, based on the principle of Homophily. At every connection attempt, the variate v is drawn uniformly from [0,1] and the link is established if , where T_h is a parameter (**homophily_strength**)
4. If the relationship from X to Y is established, then Y attempts to reciprocate and establish the relationship from Y to X, which succeeds if and only if both nodes are still available and the homophily principle is met.

APK has two kinds of homophily: direct (characterized by the parameter T_h above) and geographical. Geographical homophily refers to the tendency for similar individuals to live near each other and therefore to form a connection. APK models the geographic homophily through the encounter process described earlier, that creates the network. We validated the total effect of these processes (Figure 5).

***Removal of network connections:*** Network connections are not permanent and may be disrupted after several months, or even weeks. To model this, after each connection is formed, the software estimates the time when the connection would be severed. This lifetime is based on an exponential distribution with a mean of **mean_tie_lifetime**. Connections are also severed if one of the PWID is removed from the simulation (due to death, cessation of drug use, or emigration from the metropolis).

**Supplemental References**

1. Garfein RS, Swartzendruber A, Ouellet LJ, Kapadia F, Hudson SM, Thiede H, et al. Methods to recruit and retain a cohort of young-adult injection drug users for the Third Collaborative Injection Drug Users Study/Drug Users Intervention Trial (CIDUS III/DUIT). Drug and alcohol dependence. 2007;91 Suppl 1:S4-17. doi: 10.1016/j.drugalcdep.2007.05.007. PubMed PMID: 17582705.

2. Boodram B, Golub ET, Ouellet LJ. Socio-behavioral and geographic correlates of prevalent hepatitis C virus infection among young injection drug users in metropolitan Baltimore and Chicago. Drug and alcohol dependence. 2010;111(1-2):136-45. Epub 2010/05/18. doi: 10.1016/j.drugalcdep.2010.04.003. PubMed PMID: 20472373.

3. Huo D, Ouellet LJ. Needle exchange and injection-related risk behaviors in Chicago: a longitudinal study. J Acquir Immune Defic Syndr. 2007;45(1):108-14. Epub 2007/04/27. doi: 10.1097/QAI.0b013e318050d260. PubMed PMID: 17460474.

4. Tempalski B, Pouget ER, Cleland CM, Brady JE, Cooper HL, Hall HI, et al. Trends in the population prevalence of people who inject drugs in US metropolitan areas 1992-2007. PloS one. 2013;8(6):e64789. Epub 2013/06/12. doi: 10.1371/journal.pone.0064789. PubMed PMID: 23755143; PubMed Central PMCID: PMC3673953.

5. McPherson M, Smith-Lovin L, Cook JM. Birds of a feather: Homophily in social networks. Annual Review of Sociology. 2001:415-44.

6. Neaigus A, Friedman SR, Goldstein M, Ildefonso G, Curtis R, Jose B. Using dyadic data for a network analysis of HIV infection and risk behaviors among injecting drug users. NIDA research monograph. 1995;151:20-37. Epub 1995/01/01. PubMed PMID: 8742759.

7. Sacks-Davis R, Daraganova G, Aitken C, Higgs P, Tracy L, Bowden S, et al. Hepatitis C Virus Phylogenetic Clustering Is Associated with the Social-Injecting Network in a Cohort of People Who Inject Drugs. PloS one. 2012;7(10):e47335. doi: 10.1371/journal.pone.0047335.

**Table 1: Parameters for constructing the population database CNEP+.** Values for the parameters were extracted from the references or estimated. The ranges were assigned based on the reported variability across references and evaluation of the quality of the estimate. HR=PWID in harm reduction programs.

| **Parameter** | **Value** | **Range** | **Notes/Ref.** |
| --- | --- | --- | --- |
| mean_enrichment_Chicago | 0.5 | 0.3-0.7 | CIDUS III data ([1](#_ENREF_1)) |
| mean_enrichment_suburbs | 2 | 1.5-2.5 | expert judgment and partial data |
| nonHR_age_boost | 3 years (older than CNEP) | 1.5-4.5 | ([3](#_ENREF_3)) |
| nonHR_age_started_boost | 1.7 years (earlier than CNEP) | 0.7-2.7 | ([3](#_ENREF_3)) |
| nonHR_degree_boost | 0.24 (over CNEP cohort) | 0.14-0.34 | CIDUS III ([1](#_ENREF_1), [2](#_ENREF_2)) |
| nonHR_hcv_ab_boost | 0.011 (over CNEP) | 0-0.02 | CIDUS III([1](#_ENREF_1), [2](#_ENREF_2)) |
| nonHR_injection_ratio | 0.84 | 0.8-0.9 | ([3](#_ENREF_3)) |
| nonHR_sharing_boost | 0.33 | 0.23-0.43 | ([3](#_ENREF_3)) |

Table 2. Parameters for building the networks among PWID in APK. See Eq. 1 for details. Values for the parameters were extracted from the references or calibrated by matching to data. The ranges were assigned based on the reported variability across references and evaluation of the quality of the estimate.

| **Parameter** | **Value** | **Range** | **Notes** |
| --- | --- | --- | --- |
| homophily_strength | 0.7 | 0.6-0.8 | Validated against YSN survey (see Materials and Figure 5) |
| interaction_home_cutoff  ($d_{\max}$ in Eq. 1) | 2.0km | 1-3km | Assigned based on typical neighborhood size in Chicago. |
| interaction_rate_constant ($c_{h}$ in Eq. 1) | 0.016 per day | 0.01-0.026 | Calibrated by fitting to YSN  (Figure 5) |
| interaction_rate_at_drug_sites ($c_{d}$ in Eq. 1) | 0.0001 per day | 0.00005-0.00015 | Calibrated by fitting to YSN  (Figure 5) |
| interaction_rate_exzone ($c_{\infty}$ in Eq. 1) | 0.086 km^2^ per day | 0.076-0.096 | Calibrated by fitting to YSN  (Figure 5) |
| mean_tie_lifetime | 1.72 years | 1.0-2.5 | Estimated from ([6](#_ENREF_6)). See also ([7](#_ENREF_7)). Actual ties may have much shorter or longer existence than the population-wide mean. |
